# Seed Characteristics and Terpene Variability of Mediterranean Fir Species (*Abies nebrodensis*, *A. pinsapo*, and *A. alba*)

**DOI:** 10.3390/plants14060892

**Published:** 2025-03-12

**Authors:** Waed Tarraf, Tolga İzgü, Carla Benelli, Gabriele Cencetti, Marco Michelozzi, Alfonso Crisci

**Affiliations:** 1 Institute of BioEconomy (IBE), National Research Council (CNR), Via Madonna del Piano 10, 50019 Sesto Fiorentino, Florence, Italy; tolga.izgu@ibe.cnr.it (T.İ.); carla.benelli@ibe.cnr.it (C.B.); alfonso.crisci@ibe.cnr.it (A.C.); 2 Institute of Biosciences and Bioresources, National Research Council (IBBR), National Research Council (CNR), Via Madonna del Piano 10, 50019 Sesto Fiorentino, Florence, Italy; gabriele.cencetti@cnr.it (G.C.); marco.michelozzi@cnr.it (M.M.)

**Keywords:** Sicilian fir, European silver fir, Spanish fir, threatened species, conifers, chemotaxonomic markers, empty seeds

## Abstract

Most fir species in the Mediterranean have small to medium-sized distributions, are often endemic and endangered, and are mainly found in relict areas, except for *Abies alba*. The IUCN Red List of Threatened Species identified *Abies nebrodensis* as the rarest conifer in the world, with only 30 adult trees remaining. Additionally, *Abies pinsapo* is threatened and limited to five fragmented locations in Spain and Morocco. This study aimed to characterize the seed terpene profiles of Mediterranean *Abies* species, such as *A. nebrodensis*, *A. pinsapo*, and *A. alba*, since morphological results showed minimal variation among the *Abies* populations examined. Terpenes were extracted using n-heptane and then analyzed by GC-MS. The chemical composition revealed the dominance of limonene and α-pinene as the main monoterpenes in all the species, while *A. nebrodensis* reported the considerable presence of germacrene D-4-ol and selina-6-en-4-ol as sesquiterpenes. The relative contents of most of the terpenes were significantly different among the species, and subsequent statistical multivariate analysis showed clear discrimination among three distinct groups. These results confirmed the suitability of the terpene profile as a potential tool to study chemotaxonomic differences between species from the same family. Moreover, the compounds identified can be interesting for further studies on plant defense against biotic stress to reduce the risk of species extinction caused by pests and diseases.

## 1. Introduction

Conifers are the most important source of industrial wood, supplying over 50% of the world’s timber harvest [1]. Among them, the genus *Abies* Miller is the second largest genus in the Pinaceae family after *Pinus* [2] and is considered of major ecological and economic importance for coniferous forests. [3]. It is represented by approximately 50 species [4], and is mainly distributed in northern temperate regions of Asia, Europe, North and Central America, and North Africa [5]. Several *Abies* species are located in the mountains of the Mediterranean region while the others occupy only limited areas [6]. Based on the morphological and anatomical data and the geographic distribution, Mediterranean *Abies* species are grouped into two sections [7]. The Piceaster section features *A. pinsapo* Boiss. in southern Spain, *A. numidica* Carr. in Northwest Algeria, and *A. marocana* Trab. and *A. tazaotana* Villar in Northwest Morocco, while the *Abies* section includes *A. alba* Miller and *A. nebrodensis* (Loyac.) Mattei in Sicily [8].

Many fir species in the Mediterranean have limited geographical ranges, are often endemic and endangered, and are found in relict areas. However, *A. alba* [9] is an exception. It is the most widely distributed coniferous species in the central European forests [10]. This evergreen tree, also known as silver fir [11], can grow up to 40–50 m tall and produces cones that are 9–17 cm long and 3–4 cm wide, with about 150–200 scales [12].

Among the threatened species is *Abies pinsapo* Boiss. (Spanish fir). A relict fir endemic to Mediterranean mountain ranges in Spain and Morocco [13], it mainly exists in a limited area at five sites, three in southern Spain and two in the north of Morocco [14], at an altitude of 500–2000 m [15]. Spanish fir is found in about 3600 ha in south Spain, while it covers about 3000 ha in northern Morocco [16]. Over the centuries, this species has been intensely damaged due to unsustainable practices such as overgrazing, uncontrolled logging, and pollarding [17]. Thus, it is listed as an endangered tree by the International Union for Conservation of Nature (IUCN, https://www.iucnredlist.org/fr/species/42295/10679577; accessed on 24 February 2025), and forms pure woodlands that have special ecological importance for many organisms [18].

According to the IUCN Red List of Threatened Species, *A. nebrodensis* (Lojac.) Mattei, also known as Sicilian fir, is another conifer species and is classified as the most endangered tree in the Mediterranean region. Currently, only 30 trees remain in the Madonie Regional Park located in Northern Sicily, Italy; this endemic species is limited to an area of 84 ha, at an altitude of 1400–1650 m above sea level [19], although in the past it had a wider distribution [20]. The species is at heightened risk due to high genetic erosion, slow growth (only 50 cm in height over 10 years), and poor natural regeneration. Though it produces good seeds every 3–4, many of these seeds are empty, which leads to a low germination rate. Also, excessive grazing by wild herbivores is considered a serious problem for the growth of both adult trees and their new natural regeneration [21].

Conifers have evolved many physical and chemical defenses [22]. According to Lewinsohn et al. [23], terpenes are conifers’ most significant chemical defenses of a volatile nature, and they largely affect insect orientation and herbivory, as noted by Lundborg et al. [24]. Plants produce these substances as secondary metabolites and have a crucial protective role against stress, herbivores, and pathogenic microorganisms [25]. Forests are recognized as a major source of terpenes [26]; in particular, these volatiles are abundant and diverse in the family Pinaceae [27]. Their content and type are distinctive to each species and even vary between individual trees [28,29], and so terpenes can be a powerful tool for studying chemotaxonomic differences between species from the same family or genus [30]. Indeed, the relative percentages of constitutive terpene in mature tissue are often used in chemosystematic studies to characterize species, provenances, and clones since these compounds are under strong genetic control and are altered little by abiotic factors [31,32,33]. Further, these substances are used as ecological and biochemical markers in many coniferous taxa [34,35,36,37] to evaluate species-specific composition responses to the environment [38], to estimate the geographic variability of conifer species [39,40], and to determine trees’ resistance to pests or diseases because they are involved in the defense mechanisms of plants against insects and fungal infestations [41].

The relationships between the quantity and composition of terpenes were largely studied in coniferous species on different parts of the plant [37,38,39,40,42], and less often in seeds, to explain the chemotypes of and chemical variation in terpenes in trees of different geographical origins. In the literature, there is no information on the terpene profiles isolated from empty seeds collected from critically endangered Sicilian fir, endangered Spanish fir, and the common European silver fir species.

Therefore, this study aimed to study morphological seed features and characterize variation in seed terpene profiles of different *Abies* species grown in Mediterranean areas such as *A. nebrodensis*, *A. alba*, and *A. pinsapo.*

## 2. Results and Discussion

### 2.1. Seed Characteristics

**Figure 1:**
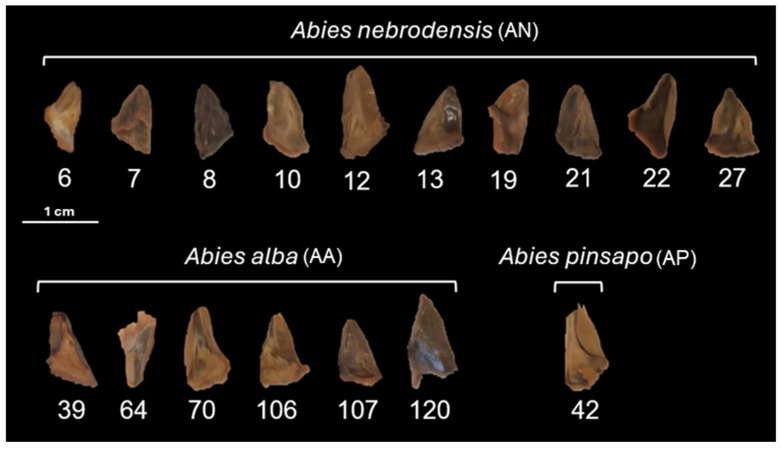
Representative empty seeds of *A. nebrodensis*, *A. pinsapo*, and *A. alba*. The numbers are the identification (ID) numbers of seeds collected from each population.

Seed features play a crucial role in species identification, classification, and understanding ecological adaptations [43]. Therefore, the moisture content (MC) percentage, 100-seed weight, length, and width of seeds from *A. nebrodensis* (AN: 6, 7, 8, 10, 12, 13, 19, 21, 22, and 27), *A. alba* (AA: 39, 64, 70, 106, and 120), and *A. pinsapo* (AP42) (Figure 1) were measured. In the present study, all the seed characters studied revealed significant variations among the species (Table 1). The highest coefficient of variation was recorded for moisture content (26.85%), followed by 100-seed weight (18.51%), seed width (15.10%), and seed length (9.12%).

The results indicated a highly significant difference in moisture content among the three *Abies* species (*p* < 0.001). The moisture content ranged from 6.66 ± 3.74% in *A. alba* to 18.99 ± 3.35% in *A. nebrodensis* (Table 1). The percentage of MC is an important seed trait that affects viability, desiccation tolerance, and storage potential. Seeds collected from Sicilian fir populations exhibited the largest variation, with MC ranging from 7.20 ± 1.61% to 18.99 ± 3.35%. In contrast, seeds from silver fir showed less variability, with moisture contents ranging from 6.66 ± 3.74% to 13.47 ± 6.42%. The significantly higher MC percentage found in *A. nebrodensis* compared to the other two species may indicate differences in seed desiccation tolerance, potentially influencing dormancy and germination strategies.

The seed dimensions, including length and width, that were recorded in *A. nebrodensis* showed significant variability, with lengths ranging from 8.7 ± 0.3 to 12 ± 1.2 mm and a width ranging from 0.49 ± 0.05 to 0.65 ± 0.1 mm (Table 1). The longest seeds were measured for *A. nebrodensis*. *A. alba* exhibited the widest seeds, while intermediate values of 10.8 mm in length and 0.59 mm in width were found in *A. pinsapo*. The differences in seed dimensions noted in our study are consistent with the findings by Singh et al. [44] for various populations of *P. kesiya.* Also, Hodžić et al. [45] indicated that there was variation among southern and northern populations of *P. heldreichii,* reporting 5.6 mm to 7.5 mm seed lengths and 3.0 mm to 4.4 mm seed widths. Mustafa et al. [46] reported seed widths between 3.07 mm and 3.71 mm in *P. halepensis*, a significantly larger size range than in *Abies*, but with similarly high intraspecific variation. Comparatively, Ghimire et al. [47] noted that *A. koreana* seeds measured 5.57 ± 0.35 mm in length and 2.92 ± 0.22 mm in width, while *A. holophylla* seeds were approximately 1 cm in length and 5 mm in width. These comparisons illustrate the extensive range of seed sizes among genotypes [48] and shapes across different species and provenances [49]. Since the seeds were collected from various locations, variances observed in seed parameters may be attributed to genetic variability and environmental conditions that occurred during seed development [50].

Significant variations were observed in the weight of 100 seeds from the investigated *Abies* species (Table 1). *A. alba* had the highest average weight at 6.42 ± 1.41 g, while the lowest weight of 2.93 ± 0.07 g was recorded in *A. nebrodensis*. A similar finding was noted by Skrzyszewska and Chłanda [49], who reported that seed weights for *A. alba* ranged from 38.92 g to 53.27 g per 1000 seeds collected from various provenances. Also, the seed weight was more influenced by individual trees, as noted by Trujillo-Ríos et al. [51]. While seed weight is commonly used in taxonomy and ecological studies due to its correlation with reproductive strategies and environmental adaptation, our results indicated no significant intraspecific or interspecific variation (Table 1).

**Figure 2:**
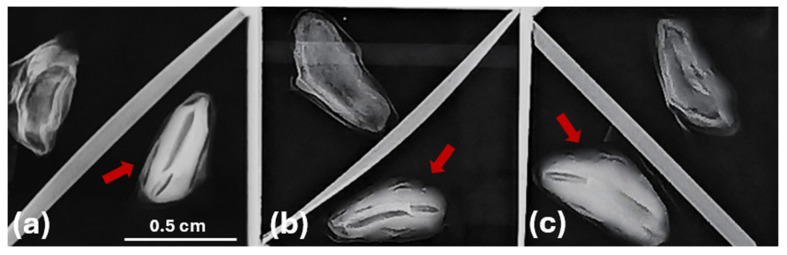
X-ray image analysis of empty and full seeds of *Abies* species, with seeds containing zygotic embryos indicated by red arrows: (**a**) *A. nebrodensis*, (**b**) *A. alba*, (**c**) *A. pinsapo*.

X-ray radiography techniques are used to estimate seed quality, mainly by assessing the development stages of seeds [49]. Their application in this study supports the selection between fully developed seeds, containing a well-formed embryo and endosperm, and empty (Figure 2) ones [52]. Such insights into internal seed characteristics are crucial for assessing seed quality and understanding the variability in seed traits observed among the populations from the three *Abies* species.

**Figure 3:**
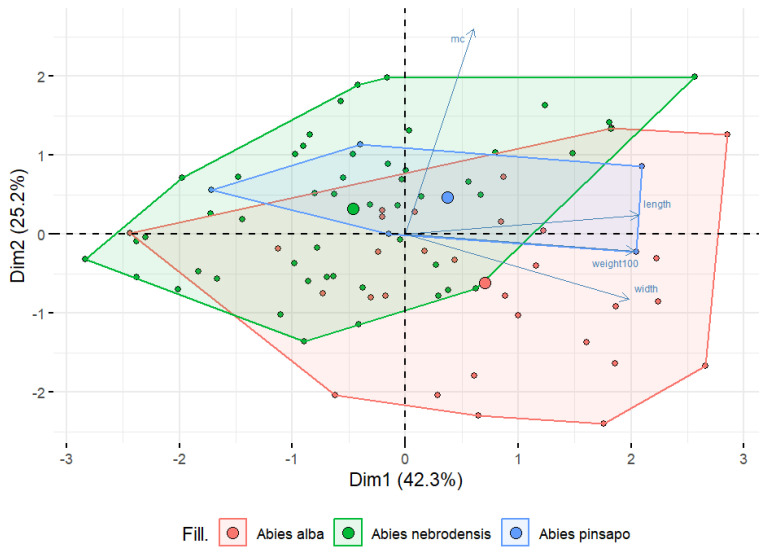
A biplot of principal component analysis based on moisture content (%) and morphological characteristics in the populations of the three *Abies* species studied.

To compare the differences among seeds from the three *Abies* species, morphological features and moisture content were analyzed using PCA, as shown in Figure 3. PCA was performed on a dataset of 17 *Abies* populations and 4 different variables to reduce the data dimensionality and to reveal the potential relationships among the parameters examined. The results showed that the first two PCs accounted for 76.5% of the total variability, explaining 42.3% for PC1 and 25.2% for PC2. However, there was overlap and a close distance among the populations, and so the tested features could not completely distinguish *A. alba*, *A. nebrodensis*, and *A. pinsapo*. From Figure 3, it can be seen that most of the samples of *A. alba* compared with the others were located on the right side of the biplot, which was positively correlated with the weight of the 100 seeds, length, and width, indicating that *A. alba* had bigger and heavier seeds than those from *A. nebrodensis* and *A. pinsapo*. Conversely, the major samples of *A. nebrodensis* were separated and located on the left side of the biplot, representing a negative correlation with all the morphological features. These observations confirm the existence of overlap among all the populations and make distinguishing seeds of one species from another quite difficult.

The morphological results indicated the existence of minimal variation among the *Abies* populations examined. In conifers, there is no external difference between empty and full seeds, although they have different weights [53]. Therefore, a further investigation into the chemical composition was conducted to rebut the similarity between seeds from various populations, since terpenes are known to correlate with geographic distributions [54].

### 2.2. Terpene Profile

**Figure 4:**
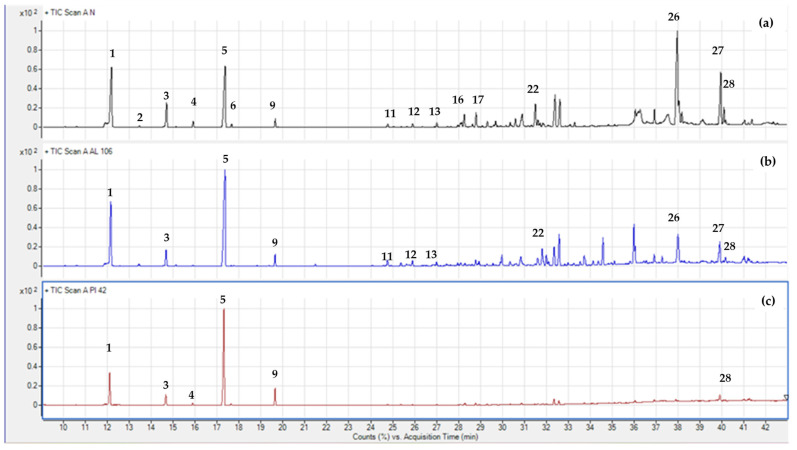
Gas chromatograms of *A. nebrodensis* (**a**), *A. pinsapo* (**b**), and *A. alba* (**c**). Compounds were identified by retention indices, mass spectra (NIST library), and standard comparisons. The compounds identified were (1) α-pinene; (2) camphene; (3) β-pinene; (4) myrcene; (5) limonene; (6) β-phellandrene; (9) terpinolene; (11) α-cubebene; (12) α-copaene; (13) β-copaene; (16) β-elemene; (17) terpinen-4-ol; (22) germacrene D; (26) germacrene D-4-ol; (27) α-epi-cadinol; (28) selina-6-en-4-ol.

In this work, three *Abies* species grown in the Mediterranean basin were analyzed by GC-MS in order to characterize the overall terpene profile. Table 2 lists the volatile compounds, given in percent (%) of the total mass area (% area pct), as mean ± standard error values. In total, 28 compounds were classified into monoterpenes hydrocarbons, oxygenated monoterpenes, sesquiterpene hydrocarbons, and oxygenated sesquiterpenes. Figure 4 shows three gas chromatograms of *A. nebrodensis* (Sicilian fir), *A. pinsapo* (Spanish fir), and *A. alba* (silver fir), indicating some characteristic volatiles with numbers. According to the mean values of terpenes in each species (Table 2), the most dominant classes in all analyzed species were monoterpene hydrocarbons [55]. There was also a considerable presence of sesquiterpene hydrocarbons, but only in the case of *A. nebrodensis*. The highest percentage of monoterpenes hydrocarbons was noted in *A. pinsapo* (92.53%) and *A. alba* (82.95%), while *A. nebrodensis* reported the lowest presence (66.52%) in favor of sesquiterpene compounds. This result concurs with the work of Wajs-Bonikowska et al. [12] and Schicchi et al. [56], where monoterpene hydrocarbons in the seeds of *A. alba* and leaves of *A., respectively,* were the most dominant chemical group. The next most represented terpene classes in *A. nebrodensis* were sesquiterpene hydrocarbons (20.59%) and oxygenated sesquiterpenes (10.61%), while the least present were oxygenated monoterpenes, found in either *A. pinsapo* (1.14%) or *A. alba* (4.47%).

The seeds from all *Abies* species are characterized by the major presence of individual monoterpenes and dominated by a large proportion of monoterpenes [42]. Table 2 shows that the seeds contained the following monoterpenes: α-pinene, β-pinene, myrcene, limonene, and trans-verbenol. The most expressed monoterpene in *Abies* seeds was limonene, followed by α-pinene; however, the abundance of these monoterpenes differed noticeably among species.

This study is the first report on the composition of empty seeds from some endangered Mediterranean firs. In *A. nebrodensis*, the major compound was limonene (49.57%), while limonene and α-pinene represented the most abundant volatiles in *A. pinsapo* (66.03 and 18.85%, respectively) and *A. alba* (62.42 and 15.43%, respectively). The presence of sesquiterpenes was limited to germacrene D-4-ol (9.27%) and selina-6-en-4-ol (9.83%) in *A. nebrodensis*, representing 19.10% of the total identified compounds. For *A. nebrodensis*, previously published results by Schicchi et al. [56] on the leaves stated that other sesquiterpene hydrocarbons like longifolene (0.1%) and δ-cadinene (<0.05%) were present in minimal amounts. In addition, β-pinene was another monoterpene compound detected in all three fir taxa, with expression ranging from 5.89% (*A. pinsapo*) to 3.05% (*A. nebrodensis*). Thus, the phytochemical profile of each species could be recognized as follows:

*A. nebrodensis*: limonene > α-pinene > selina-6-en-4-ol > germacrene-D-4-ol, representing 79.76% of the total terpenes.

*A. pinsapo*: limonene > α-pinene > β-pinene, representing 90.76% of the total known terpenes.

*A. alba*: limonene > α-pinene, representing 77.85% of the total terpenes.

The present research has identified limonene and α-pinene as the primary compounds in the seeds of *A. nebrodensis*. A previous study by Schicchi et al. [56] examined the chemical composition of the leaves and reported the absence of limonene in favor of a higher content of β-pinene (48.8%), followed by α-pinene (17.5%). The authors attributed the lack of limonene in the leaf profile to genetic factors rather than environmental conditions. Among the volatile compounds found in the seeds (Table 2), camphene was detected in a much lower concentration (0.24%), while in the essential oil from the leaves, camphene was the third most dominant terpene, with 12.7% [56]. This highlights a notable difference in terpene profiles between the seeds and leaves of *A. nebrodensis*.

Our results demonstrated that the most abundant terpenes in the seeds of silver fir were limonene (62.42%) and α-pinene (15.43%), representing 77.85% of the total identified compounds, whilst Wajs-Bonikowska et al. [12] only reported limonene (>70%) as the major volatile in the seeds of this species from Poland. Similarly, limonene dominated the chemical profile of Italian *A. alba* seeds, representing >75% of the total identified compounds [57]. The empty seeds of *A. alba* contained considerable concentrations of β-pinene and verbenone [10], at 3.21 and 2.16% (Table 2). Additionally, selina-6-en-4-ol was another terpene found in silver fir seeds (Table 2), with a small concentration of 3.16%. This finding differed from a previous study by Wajs-Bonikowska et al. [10], which reported a significantly higher percentage (51.7%) of this compound in an *A. alba* seed hydrolate.

*A. pinsapo* was another species investigated in this study. Its chemical seed profile was dominated by 66.03% limonene and 18.85% α-pinene, followed by 5.89% β-pinene and 2.55% selina-6-en-4-ol (Table 2). These are the first published data on the terpene profile of Spanish fir seeds, considering that all previous studies widely focused on the phytochemical composition of the wood. Barrero et al. [58] isolated the first sesquiterpenoids from the acid fraction of the hexane extract, while later Barrero et al. [59] obtained α-cubebene, longifolene, α-murolene, and δ-cadinene from the neutral fraction and γ-cadinene and germacrene D via the usual spectroscopic techniques. Among these compounds, only α-cubebene and germacrene D appeared as traces (0.18 and 0.42%, respectively) in this study (Table 2).

According to the literature, the primary terpenes identified in *Abies* species include α-pinene, β-pinene, camphene, limonene, and δ-3-carene [60]. However, the terpene compositions of the seeds of *A. nebrodensis* and *A. pinsapo,* growing in their natural habitats, have not been studied. In this paper, the most prevalent compounds found were limonene and α-pinene, while the amounts of β-pinene and camphene varied depending on the tested specific species. It was noted that santene and tricyclene [56], as known compounds of *A. nebrodensis*, as well as bornyl acetate [61], found in *A. pinsapo,* and δ-3-carene [62], found in *A. alba,* were completely absent from the chemical profiles of seeds (Table 2). Compared to the current study, Kshatriya et al. revealed distinct monoterpenes in the seeds of other *Abies* species [42]. α-pinene and β-myrcene, found in *A. amabilis*, α-pinene and β-pinene, found in *A. balsamea*, limonene, found in *A. grandis*, and β-phellandrene, found in *A. lasiocarpa,* were the most predominant compounds. Such variation in the chemical profiles in conifer terpenes is attributed to the complex biosynthetic systems encoded by a large number of terpene synthase (TPS) genes and known terpene-modifying cytochrome (P450) [63,64]. Thus, genetic relationships between populations can be illustrated based on the content of monoterpenes and sesquiterpenes [65].

The highest number of terpenes was found in silver fir (28), followed by Sicilian fir (27) and Spanish fir (24), and many common compounds were identified in varying ratios (Table 2). Indeed, statistical analysis revealed variation in the mean values of the detected compounds. The different terpene distributions found among the three *Abies* species were verified with the Kruskal–Wallis test, as a nonparametric method, to check if multiple groups followed the same distribution. In most cases, there were notable differences (*p* < 0.05, *p* < 0.01, and *p* < 0.001) in the proportions of volatile compounds according to the fir species (Table 2).

Statistically significant differences were found in most of the detected monoterpenes and sesquiterpenes, and only 3 (α-pinene, limonene, and boranyl acetate) out of 25 compounds showed no statistically differences between species (Table 2). For example, the contents of germacrene D-4-ol (9.27%) and selina-6-en-4-ol (9.83%) in *A. nebrodensis* were greater compared to *A. pinsapo* (0.16 and 2.55%, respectively) and *A. alba* (0.69 and 3.16%, respectively), whereas verbenone was more abundant in *A. alba* (2.16%) than in *A. nebrodensis* (0.43%) and *A. pinsapo* (0.01%).

To determine which groups contribute to significant differences, we employed Dunn’s multiple comparisons as the post hoc procedure for each statistically significant variable. Dunn’s tests showed the highest number of nineteen differing compounds between *A. nebrodensis* and *A. alba*, whereas there were sixteen differing compounds each between *A. nebrodensis* and *A. pinsapo*, as well as between *A. alba* and *A. pinsapo* (Table 3). The most frequent terpenes that showed significant differences between each analyzed pair species were α-pinene, camphene, myrcene, *p*-cymene, verbenone, and α-copaene. However, terpinolene, bornyl acetate, and trans-verbenol were the only terpenes with no significantly different values for the three analyzed groups.

Terpene profiles were studied as biochemical markers in coniferous trees from various geographical origins to define chemotypes and explain their diversity [66]. Indeed, notable variation is reported in species from the genus *Abies*, corresponding to whether the individuals are grown naturally in their native habitat or artificially cultivated [60]. β-pinene was the most abundant compound in the natural populations of *Abies* species from Romania, Serbia, North Macedonia, and Bulgaria, followed by limonene-β- phellandrene [37] or α-pinene and camphene [67]. On the other hand, α-pinene was the main volatile in the *A. cephalonica* population from Greece [37,67]. Bornyl acetate was found in the artificial plantations of *A. alba* [61], and δ-3-carene prevailed in the naturally distributed *A. nordmanniana* from Turkey [62].

Many studies have demonstrated that monoterpenes are effective tools for chemotaxonomic surveys, helping to determine intraspecific variation and define certain species [35,37,38]. To overcome the challenges associated with classifying species based solely on morphological data, it was advised to use gas chromatography–mass spectrometry (GC-MS) techniques to identify potential terpene markers with multivariate analyses [68].

**Figure 5:**
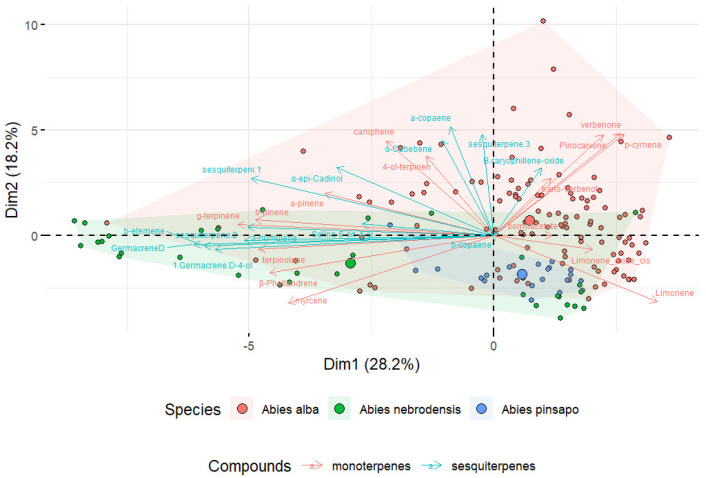
Principal component analysis (PCA) biplot of three *Abies* species based on the variance in the terpene compounds.

For this reason, PCA of matrix data was performed to investigate the variability among the three *Abies* species. The first and second components of the PCA of the collected terpenes explained 46.4% of the variation in the data, 28.2% of the variation in PC1, and 18.2% of the variation in PC2 (Figure 5). Moreover, as shown in the PCA biplot, the *Abies* had different terpenoid profiles that could be visually distinguished according to the species. The length of the arrow indicates the contribution of each compound to the first two components in the PCA. Longer arrows show a higher contribution by the variables, while shorter arrows denote a lower contribution by the variables. This is a useful tool for data reduction to visualize the similarities among complex datasets. An acceptable degree of separation was found between the samples from *A. nebrodensis*, *A. alba*, and *A. pinsapo*, suggesting that the different terpenes contribute to the distinctive terpene profile of *Abies* samples. Although the results of PCA depicted the separation of the *Abies* species encouraged to some extent, it is important to note that PCA only provides visual information, with no specific index to verify the actual differences.

**Figure 6:**
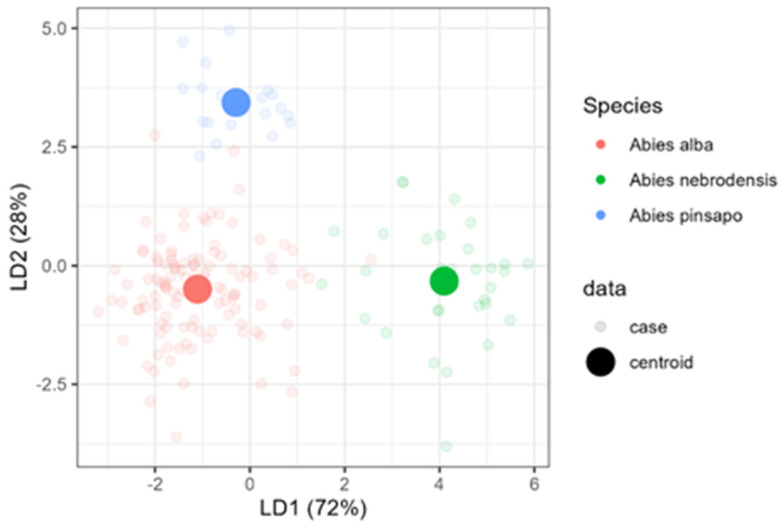
Linear discriminant analysis (LDA) for the classification of three *Abies* species.

Therefore, to build a tool for *Abies* species classification using a relative terpene abundance, linear discriminant analysis (LDA) was used on the selected data matrix. The previous data were chosen to avoid collinearity, as mentioned in the Materials and Methods Section, and the subsequent compounds, β-elemene, germacrene D, and germacrene-D-4-ol, were excluded. In Figure 6, an LDA biplot of data with species centroids is presented. The LD1 and LD2 functions explained 96.30% of the total variance (Wilks’ λ = 0.086134, *p* < 2.2 × 10^−16^). LDA, unlike PCA, is a supervised ordination method that maximizes the separation among the data classes and minimizes distance within the class. LDA separated all three species well; indeed, *A. alba* and *A. nebrodensis* were discriminated by LD1, while LD2 enabled the separation of *A. pinsapo* from the other two species (Figure 6).

LDA exhibited more sharp classes among the 25 compounds. LDA was also convenient for isolating *A. nebrodensis* from others. *A. pinsapo* was also more isolated away from *A. alba* and *A. nebrodensis*. Only a few cases of *A. alba* seemed close to *A. nebrodesis* and *A. pinsapo*. The first two discriminant functions effectively discriminated between *Abies* species (Table 4); therefore, three groups representing each species were clearly observed. The first discriminant function explained 72% of the total variance and the variables with the greatest discriminant ability were sesquiterpene.1 (2.37) and *p*-cymene (2.11). The second discriminant function accounted for 28% of the data variation and was mainly correlated with *p*-cymene (1.85) and pinocarvone (1.79).

*Abies* is one of the most explored genera for chemotaxonomic purposes [69]. Earlier, Otto and Wilde [70] marked the importance of terpene profiles to differentiate species and subspecies. In this context, α-pinene, β-pinene, bornyl acetate, and *p*-cymene are the most characteristic monoterpenes that may serve as chemosystematic markers in conifers. Thus, the separation of the three *Abies* species was confirmed by analyzing their terpene compositions. Not all compounds provide the same level of discrimination, but the most effective ones in differentiating between species include *p*-cymene and sesquiterpene.1, as well as terpinolene and pinocarvone. Our results indicate that sesquiterpenes could be more involved in species diversity than monoterpenes [71], even though their quantities are relatively low compared to the total identified composition.

**Figure 7:**
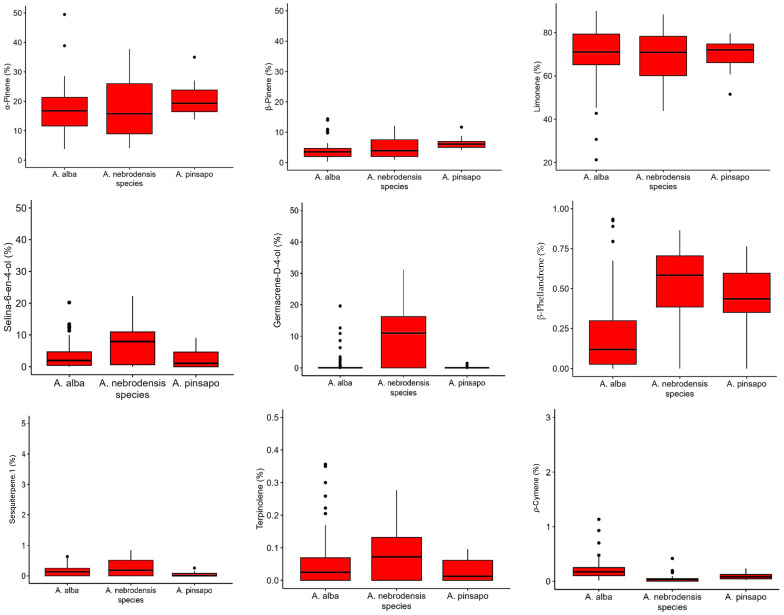
The variation in the relative percentage of various terpenes detected in the empty seeds of *A. nebrodensis*, *A. alba*, and *A. pinsapo* grown in the Mediterranean basin.

The box plots of the most important terpenes are shown in Figure 7. It was found that the relative contents of α-pinene, β-pinene, limonene, selina-6-en-4-ol, germacrene-D-4-ol, β-phellandrene, sesquiterpene.1, and terpinolene were higher in *A. nebrodensis*. The relative percentages of α-pinene and β-pinene in *A. pinsapo* were greater than those in *A. alba*. Moreover, the relative content of *p*-cymene was greater in *A. alba* than in other species, while the levels of sesquiterpene.1 was higher in *A. alba* and *A. nebrodensis* than *A. pinsapo*. The key biomarker for *A. nebrodensis* was germacrene-D-4-ol, and the key biomarker for *A. alba* was *p*-cymene.

Generally, longifolene, δ-cadinene, and α-humulene were among the sesquiterpenes most commonly used to discriminate *Abies* species, particularly those grown in the Mediterranean basin [65]. The comparison of our results with those of previous studies pointed out that not only could limonene and α-pinene be included in the chemotaxonomic research of the genus *Abies*, but so could others like camphene [72], β-pinene [73], β-phellandrene [74], and *p*-cymene [68].

To test the success of LDA in classifying each object in the group, classification accuracy was calculated by comparing the correctly classified samples to the total number of samples in each *Abies* species. In this context, the confusion matrix indicates that applying LDA to the complete dataset resulted in a recognition percentage of 96.97%. As shown in Table 5, LDA has acceptable classification accuracy for new samples regarding their geographical origins. The best results were reported for *A. nebrodensis* and *A. pinsapo* since all samples were properly classified with 100% accuracy. The classification accuracy of *A. alba* samples was acceptable (95.83%), as only one sample out of twenty-three was misclassified as an *A. nebrodensis* sample.

## 3. Materials and Methods

### 3.1. Plant Material

The current study was conducted on seeds from mature *Abies* spp.—trees. Seeds of *Abies nebrodensis* and *Abies alba* were collected from different sites, as detailed in Table 6, while those of *Abies pinsapo* were provided by the Department of Agriculture, Food, Environment and Forestry (DAGRI), University of Florence, Italy. All seeds were subjected to X-ray analysis to separate the full (with embryos) seeds from the empty seeds [52], and only the empty seeds were used for further investigation in this study.

### 3.2. Seed Moisture Content and Morphological Features

After X-ray analysis, 25 empty seeds (with wings removed) were used for each tree for morphometric measurements. A stereomicroscope and camera system (Zeiss Stemi 2000 C, Jena, Germany) were used for size measurements, and ImageJ software (Rasband, W.S., ImageJ, U.S. National Institutes of Health, Bethesda, MD, USA) version 1.54g; java 1.8.0-172, was utilized to measure the width and length of the photographed seeds. Weight measurements were performed using a precision balance, with an accuracy of 0.0001 g. For the moisture content analysis of the seeds, 5 seeds (0.5 g) were used for each species, and their moisture content was determined with a Moisture Analyzer (Mettler-Toledo AG, Laboratory & Weighing Technologies, Greifensee, Switzerland). All the measured values, given in millimeters, are reported as the mean and standard deviation, as shown in Table 1.

### 3.3. Terpene Isolation

Terpenes were isolated from *A. nebrodensis*, *A. alba*, and *A. pinsapo* seeds. For each plant of *Abies* species, 20 empty seeds were individually analyzed. The extractions were performed in 2 mL glass vials containing one seed with 0.2 mL of sample, followed by sonication for 30 min (3 cycles of 10 min) using an Ultrasonic cleaner (5300i EP S3, Soltec, Milan, Italy). Subsequently, samples were placed on an incubating shaker (Thermoshake, Gerhardt, Milan, Italy) at a speed of 100 rpm for 24 h at 35 °C. The next day, a fraction of 100 µL of each seed extract was transferred to GC vials and stored at 4 °C until further analysis.

### 3.4. Gas Chromatography–Mass Spectrometry (GC-MS) Analysis

Chemical analyses were carried out using an Agilent 7820A gas chromatograph coupled to an Agilent mass spectrometer 5977E (Agilent, Palo Alto, CA, USA) with a quadruple mass selective detector (electron ionization) equipped with a data processor: we employed Mass hunter Quantitative version B 07 01 and Qualitative version B 06 00. Terpenes were separated on a capillary column known as DB-Wax Ultra Inert (Agilent, 0.25 mm × 60 m × 0.5 µm film thicknesses). The following conditions were adopted: splitless (1 min); a flow of 1.2 mL min^−1^; and Helium 5.5 as a carrier gas. Overall, 1 µL of extract was injected using Gerstel MPS2 XL autosampler (Gerstel, Mülheim an der Ruhr, Germany); the injector temperature was set to 250; the initial temperature was 40 °C. It was programmed to 200 °C at 5 °C min^−1^, and then increased to 240 °C at 10 °C min^−1^ (held 5 min). The total run time was set to 44 min for each sample. The mass spectrometer operated with an electron ionization of 70 eV in scan mode in the *m*/*z* range of 29–330, at 4.5 scans s^−1^. The compounds were identified based on matching their mass spectra with those obtained from the NIST 11 (National Institute of Standards and Technology, Gaithersburg, MD, USA) library, using standards when possible, and the Kovats index.

### 3.5. Statistical Analysis

**Figure 8:**
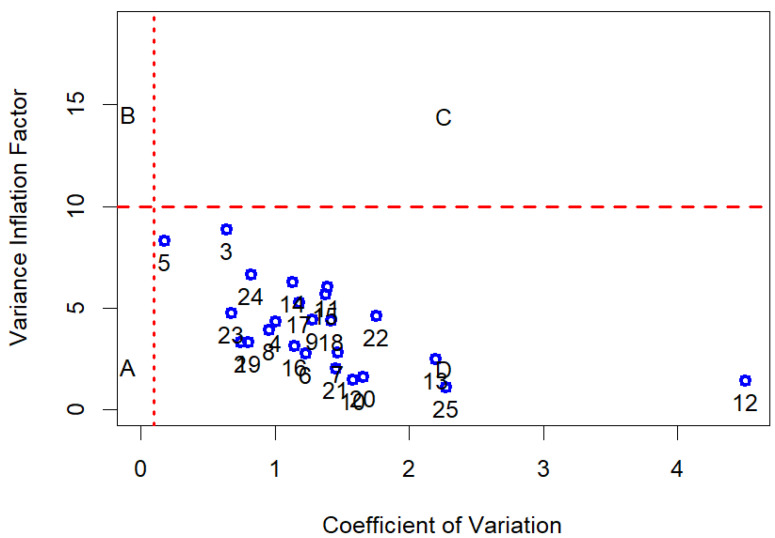
The index of Variance Inflation Factor for each single variable. Letters are labels for the areas identified by VIF (<10) and coefficient of variation (CV = 0.1) indicating the contribution of each predictor to VIF.

A one-way ANOVA test was performed for the moisture content (%) and morphological traits of empty seeds to determine significant differences in the means of the three *Abies* species. The statistical analysis of the data matrix (N = 130 observation for 28 terpenes) was carried out in 5 steps. (i) GC data were normalized as monoterpenes (C10) in relation to the total amount of monoterpene compounds, while sesquiterpenes (C15) were normalized on the sum of mono and sesquiterpenes. (ii) The second step was to verify normality, for each compound investigated, by using the Shapiro normality test [75] and the level of collinearity. The measure of the latter is given by the index of Variance Inflation Factor (VIF < 10, Figure 8) for each individual variable [76]. The variable selection to reduce collinearity was carried out using the “multiColl” R package [77] in order to perform the next step of linear discriminant analysis (LDA). (iii) The third step of the analytic framework was to evaluate if the grouping variable, represented by the tree species (*A. alba*, *A. nebrodensis*, and *A. pinsapo*), had shown a significant difference in each terpene’s relative content. The Kruskal–Wallis test was performed on the single terpene’s data and the related post hoc results were computed (Dunn test). The Kruskal–Wallis test is a non-parametric test, assumes no particular distribution of data, and is analogous to one-way analysis of variance (ANOVA). The results were presented in tabular form. (iv) A preliminary exploration of dataset variability was conducted effectively through PCA (principal component analysis). (v) Finally, a supervised classification was carried out using Fisher Linear Classification Analysis. LDA is a set of methods used in multivariate statistics to find a linear combination of features that characterize or separate two or more classes of objects [78] and is a tool for classification, dimension reduction, and data visualization. The training and test datasets needed to perform LDA were obtained by splitting the original dataset into two subsets thanks to the “createDataPartition” function of the R “caret” package [79]. The percentage of data used for training was equal to 80%, and the remaining data were used to test the classifier. The confusion matrix was performed on the test dataset. Overall statistics of classifier and accuracy statistics by species (Sensitivity or True Positive Rate, Specificity or False Negative Rate, the Detection Rate, and other classification errors) were presented. All analyses were conducted in the R statistical computing environment (version 4.3.0) by using the following R packages: we used “ggplot2” [80] for graphical purposes; we used “FactoMineR” [81] and “ordr” [82] for PCA and LDA plottings; “caret” to establish the accuracy statistics of an LDA classifier; “matrixTests” [83] provides the functions to test one-way stratification significance by species (the non-parametric Kruskal–Wallis test for terpene data and one-way ANOVA for seed data); and the “PMCMRplus” [84] and “postHoc” [85] were used for post hoc comparison. All data and codes are available at the web repository of the work: https://github.com/bioeconomy/mediterranean_fir_analisys; accessed on 24 February 2025).

## 4. Conclusions

The current study demonstrates variation in moisture content, the weight of 100 empty seeds, and seed dimensions among and within *Abies* populations. Although PCA results indicated overlapping among all the populations, making species distinction quite difficult, seed morphological traits can serve as reliable taxonomic markers in species identification and classification. These findings suggest the need for other work using additional parameters such as cone dimensions, the cone mass number of seeds per cone, etc., to establish the differences among and within the conifer species.

To address the difficulties of classifying species solely based on morphological data, chemical profiles obtained through GC/MS analysis, combined with multivariate techniques, proved effective for classifying and predicting the potential origin of unknown *Abies* samples. While the results of PCA indicated some separation among *Abies* species, its effectiveness for classification was limited by the absence of a specific index to confirm actual differences. In this context, LDA, as a supervised method, was particularly useful in separating the *Abies* species into *A. nebrodensis*, *A. pinsapo*, and *A. alba*.

The current findings from the analysis of seed terpene composition will enable further research on how different terpene profiles influence various biological processes. These processes include protecting seeds against pathogens, herbivores, and desiccation, as well as effects on seed viability and germination.

## Figures and Tables

**Table 1:** Moisture content (%) and morphological traits of empty seeds from different populations of *Abies* species.

Species	Population ID	MC (%)	Weight of 100 Seeds (g)	Length (mm)	Width (mm)
*A. alba*	AA39	7.06 ± 2.23 e	5.27 ± 1.25 b	11.5 ± 1.8 ab	0.7 ± 0.18 ab
*A. alba*	AA64	9.34 ± 3.59 de	3.73 ± 0.20 bdc	10.3 ± 1.3 abc	0.55 ± 0.13 ab
*A. alba*	AA70	6.66 ± 3.74 e	4.46 ± 0.78 bcd	10.7 ± 0.7 ab	0.63 ± 0.07 ab
*A. alba*	AA106	12.95 ± 1.92 abcde	5.13 ± 0.5 b	11.5 ± 0.9 ab	0.62 ± 0.09 ab
*A. alba*	AA107	13.47 ± 6.42 abcde	6.42 ± 1.41 a	11.4 ± 1.4 ab	0.67 ± 0.14 ab
*A. alba*	AA120	11.04 ± 1.69 bcde	4.18 ± 0.15 bcd	10.8 ± 0.7 ab	0.72 ± 0.07 a
*A. pinsapo*	AP42	14.57 ± 2.05 abcd	4.79 ± 1.56 bc	10.8 ± 0.9 ab	0.59 ± 0.09 ab
*A. nebrodensis*	AN6	13.11 ± 3.71 abcde	3.06 ± 0.25 d	11.1 ± 0.6 ab	0.57 ± 0.06 ab
*A. nebrodensis*	AN7	10.08 ± 3.03 cde	3.97 ± 0.39 bcd	9.9 ± 0.5 abc	0.54 ± 0.05 ab
*A. nebrodensis*	AN8	12.34 ± 2.07 abcde	3.11 ± 0.51 d	8.7 ± 0.3 c	0.49 ± 0.03 b
*A. nebrodensis*	AN10	12.43 ± 3.13 abcde	3.79 ± 0.22 bcd	10.3 ± 0.7 abc	0.58 ± 0.07 ab
*A. nebrodensis*	AN12	18.99 ± 3.35 a	4.93 ± 1.45 b	12 ± 1.2 a	0.62 ± 0.12 ab
*A. nebrodensis*	AN13	12.69 ± 2.62 abcde	3.97 ± 0.32 bcd	9.8 ± 0.7 bc	0.64 ± 0.07 ab
*A. nebrodensis*	AN19	7.20 ± 1.61 e	3.23 ± 0.53 cd	9.6 ± 0.5 bc	0.52 ± 0.05 ab
*A. nebrodensis*	AN21	17.36 ± 3.9 abc	3.89 ± 0.29 bcd	10.4 ± 0.2 abc	0.64 ± 0.02 ab
*A. nebrodensis*	AN22	16.97 ± 4.35 abc	2.93 ± 0.07 d	11.5 ± 1 ab	0.65 ± 0.1 ab
*A. nebrodensis*	AN27	14.98 ± 3.7 abcd	3.81 ± 0.26 bcd	9.7 ± 0.7 bc	0.62 ± 0.07 ab
*p*-value		0.0000 ***	0.0000 ***	0.0000 ***	0.0109 *
C.V%		26.85%	18.51%	9.12%	15.10%
L.S.D 0.05		4.21	0.97	0.12	0.12

values (mean ± standard deviations), with different letters in the same column significantly different according to the Student–Newman–Keuls Test. * *p* < 0.05; *** *p* < 0.001. C.V: coefficient of variation; L.S.D: least significant difference.

**Table 2:** The terpene compounds of three *Abies* species, their mean relative percentage with SE and significance (*p*), and outcomes of Kruskal–Wallis test.

No.	Terpene Compound	*A. nebrodensis*	*A. pinsapo*	*A. alba*	H Statistic	*p*-Value	Level of Significance
		n = 33	n = 20	n = 117	n = 170		
1	α-Pinene	11.09 ± 0.92	18.85 ± 0.96	15.43 ± 0.76	3.45	0.18	ns
2	Camphene	0.24 ± 0.03	0.13 ± 0.01	0.39 ± 0.02	33.05	0.00	***
3	β-Pinene	3.05 ± 0.31	5.89 ± 0.32	3.21 ± 0.19	24.45	0.00	***
4	Myrcene	1.77 ± 0.13	0.91 ± 0.09	0.66 ± 0.08	64.66	0.00	***
5	Limonene	49.57 ± 4.14	66.03 ± 1.99	62.42 ± 1.3	0.80	0.67	ns
6	β-Phellandrene	0.61 ± 0.08	0.41 ± 0.04	0.22 ± 0.03	51.26	0.00	***
7	γ-Terpinene	tr	tr	tr			
8	*p*-Cymene	tr	0.09 ± 0.01	0.18 ± 0.01	56.58	0.00	***
9	Terpinolene	0.05 ± 0.01	tr	tr			
10	Limonene oxide cis	0.14 ± 0.03	0.22 ± 0.04	0.44 ± 0.06	9.93	0.01	**
11	Pinocarvone	0.12 ± 0.07	0.27 ± 0.09	0.62 ± 0.06	29.65	0.00	***
12	Bornyl acetate	0 ± 0	0 ± 0	0.05 ± 0.02	4.78	0.09	ns
13	Terpinen-4-ol	tr	0 ± 0	tr			
14	Trans-verbenol	1.65 ± 0.32	0.88 ± 0.16	1.62 ± 0.13	10.04	0.01	**
15	Verbenone	0.43 ± 0.25	tr	2.16 ± 0.2	54.88	0.00	***
16	β-Elemene	0.8 ± 0.15	0 ± 0	0.07 ± 0.02	-	-	
17	Sesquiterpene.1	0.27 ± 0.05	0.05 ± 0.02	0.16 ± 0.01	12.62	0.00	**
18	Sesquiterpene.2	2.5 ± 0.26	1.11 ± 0.15	0.78 ± 0.11	41.27	0.00	***
19	α-Humulene	1.07 ± 0.17	0.23 ± 0.05	0.32 ± 0.04	11.62	0.00	**
20	Germacrene D	2.52 ± 0.44	0.18 ± 0.06	0.37 ± 0.08	-	-	
21	Sesquiterpene.3	0.72 ± 0.14	0.79 ± 0.19	2.33 ± 0.13	42.97	0.00	***
22	β-Caryophyllene oxide	0.3 ± 0.2	0 ± 0	1.02 ± 0.12	31.13	0.00	***
23	Germacrene D-4-ol	9.27 ± 1.68	0.16 ± 0.09	0.69 ± 0.24	-	-	
24	α-epi-cadinol	1.2 ± 0.34	0.17 ± 0.08	0.97 ± 0.11	10.94	0.00	**
25	Selina-6-en-4-ol	9.83 ± 2.59	2.55 ± 0.67	3.16 ± 0.33	13.04	0.00	***
26	α-Cubebene	0.65 ± 0.09	0.42 ± 0.07	1.07 ± 0.06	28.00	0.00	***
27	α-Copaene	0.48 ± 0.07	0.22 ± 0.05	0.79 ± 0.05	26.53	0.00	***
28	β-Copaene	1.58 ± 0.74	0.4 ± 0.07	0.75 ± 0.07	18.71	0.00	***
Monoterpenes hydrocarbons		66.52	92.53	82.95			
Oxygenated monoterpenes		2.20	1.14	4.47			
Sesquiterpene hydrocarbons		20.59	2.88	5.84			
Oxygenated sesquiterpenes		10.61	3.40	6.65			
Total identified [%]		99.92	99.95	99.91			

n: number of samples. tr—trace amount < 0.05%. Level of significance (ns: not significant, ** *p* < 0.01, *** *p* < 0.001) assessed by Kruskal–Wallis test (degrees of freedom = 2).

**Table 3:** Terpene compounds revealed statistically significant differences (indicated with asterisks) between *Abies* species (* *p* < 0.05, ** *p* < 0.01, *** *p* < 0.001); *p* values from post hoc analysis with Dunn’s multiple comparisons test (n = 2).

Compound/Species	*A. nebrodensis* vs. *A. alba*	*A. alba* vs. *A. pinsapo*	*A. nebrodensis* vs. *A. pinsapo*
α-Pinene	0.0072228 **	0.009428 **	1.08 × 10^−5^ ***
Camphene	0.0039926 **	3.212 × 10^−8^ ***	2.41 × 10^−2^ *
β-Pinene	1	4.142 × 10^−8^ ***	1.92 × 10^−6^ ***
Myrcene	1.59 × 10^−8^ ***	0.020705 *	3.83 × 10^−2^ *
Limonene	0.036737 *	0.921246	2.68 × 10^−2^ *
β-Phellandrene	1.113 × 10^−8^ ***	0.000128 ***	1
γ-Terpinene	0.13746	1	0.4414
*p*-Cymene	6.92 × 10^−14^ ***	0.010214 *	1.50 × 10^−2^ *
Terpinolene	0.72723	1	4.41 × 10^−1^
Limonene oxide cis	0.0004826 ***	1	1.05 × 10^−1^
Pinocarvone	7.108 × 10^−7^ ***	0.058621	3.28 × 10^−1^
Bornyl acetate	0.1989	0.40403	1.00
Terpinen-4-ol	0.5432794	0.0108495 *	1.91 × 10^−3^ **
Trans-verbenol	1	0.052235	2.09 × 10^−1^
Verbenone	3.691 × 10^−8^ ***	4.016 × 10^−8^ ***	1
β-Elemene	2.97 × 10^−7^ ***	0.38228	1.65 × 10^−6^ ***
Sesquiterpene.1	0.8285204	0.006418 **	2.18 × 10^−3^ **
Sesquiterpene.2	1.99 × 10^−6^ ***	0.023123 *	1.30 × 10^−1^
α-Humulene	0.0040646 **	1	4.67 × 10^−2^ *
Germacrene D	3.811 × 10^−5^ ***	1	1.81 × 10^−2^ *
Sesquiterpene.3	2.31 × 10^−9^ ***	6.179 × 10^−6^ ***	1
β-Caryophyllene oxide	8.853 × 10^−5^ ***	3.206 × 10^−5^ ***	1
Germacrene D-4-ol	3.516 × 10^−8^ ***	1	2.00 × 10^−4^ ***
α-epi-cadinol	1	0.0027414 **	4.72 × 10^−2^ *
Selina-6-en-4-ol	0.0040008 **	1	9.92 × 10^−3^ **
α-Cubebene	0.0013365 **	8.4424 × 10^−7^ ***	1.56 × 10^−1^
α-Copaene	0.011587 *	6.545 × 10^−7^ ***	4.71 × 10^−2^ *
β-Copaene	0.2532145	0.0040231 **	2.46 × 10^−4^ ***

**Table 4:** Coefficients of linear discriminants of *Abies* species in relation to terpene compounds. Significant coefficients are in bold.

Variable	LD1	LD2
α-Pinene	−0.004277661	−0.085274092
Camphene	0.143528773	−2.932279547
β-Pinene	−0.11926859	0.793177699
Myrcene	1.293628722	−0.247687015
Limonene	−0.01197199	0.024988731
β-Phellandrene	1.473550949	−0.437051156
γ-Terpinene	−1.69750848	0.410111115
*p*-Cymene	2.112028567 *	1.848104132 *
Terpinolene	−20.41296551	−4.298545602
Limonene-oxide-cis	−0.075998513	−0.608828993
Pinocarvone	−0.66069519	1.797811335 *
Bornyl acetate	0.840008653	0.478153842
Terpinen-4-ol	−0.29230847	0.134471116
Trans-verbenol	0.436510957	−0.321339896
Verbenone	−0.018931995	−0.166428292
Sesquiterpene.1	2.365264726 *	−1.590761873
Sesquiterpene.2	0.293305148	0.895506246
α-Humulene	1.283763095	−1.696687733
Sesquiterpene.3	−0.191231726	−0.506511034
β-Caryophyllene-oxide	0.071466501	−0.056933295
α-epi-cadinol	0.041705135	−0.009005742
Selina-6-en-4-ol	−0.040522488	0.04171892
α-Cubebene	−0.624629032	−0.323550455
α-Copaene	0.215010018	−0.019346339
β-Copaene	0.076285341	−0.025504718

* = largest coefficient.

**Table 5:** The confusion matrix of the results obtained for each species on the testing dataset using trained LDA classification model. Correct identifications are emphasized with bold font.

	*A. alba*	*A. nebrodensis*	*A. pinsapo*
*A. alba*	**23** (95.83%)	1 (4.17%)	-
*A. nebrodensis*	-	**5** (100%)	-
*A. pinsapo*	-	-	**4** (100%)
Total samples = 33 Total correct classifications: 32 samples = 96.97%			

**Table 6:** Location and geographical characteristics of Mediterranean *Abies* spp. populations.

Plant N°	Species	Population ID	Collection Site	Province	Altitude (m a.s.l.)	Latitude N	Longitude E	Region	Country
1	*A. alba*	AA39	Serra San Bruno-Monte Pecoraro	Vibo Valentia	1100–1400	38°31′59″	16°19′59″	Calabria	Italy
2	*A. alba*	AA107	Serra S. Bruno S. Maria	Vibo Valentia	800–1100	38°33′39″	16°18′58″	Calabria	Italy
3	*A. alba*	AA70	Vallombrosa	Florence	800–1200	43°44′58″	11°33′21″	Tuscany	Italy
4	*A. alba*	AA64	Abetone	Pistoia	1200–1500	44°08′02″	10°40′17″	Tuscany	Italy
5	*A. alba*	AA106	Serra San Bruno-Archiforo	Vibo Valentia	900–1300	38°31′59″	16°19′59″	Calabria	Italy
6	*A. alba*	AA120	Gariglione	Catanzaro	1400–1700	39°08′12″	16°38′34″	Calabria	Italy
7	*A. pinsapo*	AP42	Unknown						Spain
8	*A. nebrodensis*	AN6	Monte Scalone	Palermo	1644	37°50′25″	14°01′22″	Sicily	Italy
9	*A. nebrodensis*	AN7	Pendici di Monte Scalone	Palermo	1587.07	37°50′27″	14°01′30″	Sicily	Italy
10	*A. nebrodensis*	AN8	Pendici di Monte Scalone	Palermo	1562.33	37°50′28″	14°01′29″	Sicily	Italy
11	*A. nebrodensis*	AN10	Monte Scalone	Palermo	1509.05	37°50′34″	14°01′6″	Sicily	Italy
12	*A. nebrodensis*	AN12	Pendici di Monte Scalone	Palermo	1587.12	37°50′26″	14°01′25″	Sicily	Italy
13	*A. nebrodensis*	AN13	Pendici di Monte Scalone	Palermo	1561.5	37°50′29″	14°01′30″	Sicily	Italy
14	*A. nebrodensis*	AN19	Vallone Madonna degli Angeli e Monte Scalone	Palermo	1468.74	37°50′35″	14°01′19″	Sicily	Italy
15	*A. nebrodensis*	AN21	Vallone Madonna degli Angeli	Palermo	1402.3	37°50′50″	14°01′20″	Sicily	Italy
16	*A. nebrodensis*	AN22	Vallone Madonna degli Angeli	Palermo	1390.99	37°50′43″	14°01′15″	Sicily	Italy
17	*A. nebrodensis*	AN27	Vallone Madonna degli Angeli	Palermo	1579.08	37°50′33″	14°01′37″	Sicily	Italy

## Data Availability

Data are contained within the article.
